# Using Patient Completed Screening Tools to Predict Risk of Malnutrition in Patients With Inflammatory Bowel Disease

**DOI:** 10.1093/crocol/otab043

**Published:** 2021-07-07

**Authors:** Lorian M Taylor, Tannaz Eslamparast, Kamal Farhat, Karen Kroeker, Brendan Halloran, Nusrat Shommu, Ankush Kumar, Quinn Fitzgerald, Leah Gramlich, Juan G Abraldes, Puneeta Tandon, Maitreyi Raman

**Affiliations:** 1 Department of Medicine, University of Calgary, Calgary, Alberta T2N 4N1, Canada; 2 Department of Medicine, University of Alberta, Zeidler Ledcor Centre, 130 University Campus, Edmonton, Alberta T6G 2X8, Canada; 3 Division of Gastroenterology, University of Alberta, Zeidler Ledcor Centre, 130 University Campus, Edmonton, Alberta T6G 2X8, Canada; 4 Department of Family Medicine, University of Calgary, Health Sciences Center, 3330 Hospital Drive Northwest, Calgary, Alberta T2N 4N1, Canada; 5 Department of Medicine, Royal Alexandra Hospital, University of Alberta, Edmonton, Alberta, Canada; 6 Division of Gastroenterology and Hepatology, University of Calgary, 6D26, Teaching Research and Wellness Building, 3280 Hospital Drive NW, Calgary, Alberta T2N 4N1, Canada

**Keywords:** inflammatory bowel disease, malnutrition, nutrition screening tools

## Abstract

**Background:**

Malnutrition is associated with adverse clinical outcomes in patients with inflammatory bowel disease (IBD), however, malnutrition screening is not routinely performed. This study aimed to identify the prevalence of malnutrition in patients with IBD and compare the accuracy of patient completed screens to a gold-standard malnutrition assessment tool: the dietitian-completed subjective global assessment (SGA).

**Methods:**

This cross-sectional study was conducted at 2 hospitals and 2 ambulatory care clinics in Alberta, Canada. Patients with IBD completed 4 malnutrition screening tools: abridged patient-generated SGA (abPG-SGA), Malnutrition Universal Screening Tool (MUST), Canadian Nutrition Screening Tool (CNST), and Saskatchewan IBD–nutrition risk (SaskIBD-NR). Risk of malnutrition was calculated for each tool and differences were compared between IBD subtype and body mass index (BMI) categories. Sensitivity and specificity, negative and positive predictive values (NPV and PPV), and area under the receiver operating characteristic curve (AUC) were calculated compared to SGA.

**Results:**

Patients with Crohn’s disease (*n* = 149) and ulcerative colitis (*n* = 96) participated in this study. Overall prevalence of malnutrition using SGA was 23% and malnutrition risk for CNST, abPG-SGA, SaskIBD-NR, and MUST was 37%, 36%, 36%, and 27%, respectively. Overall, the abPG-SGA had the highest sensitivity (83%), PPV (53%), and NPV (94%), and largest AUC (0.837) compared to SGA. For patients with a BMI ≥25 kg/m^2^, sensitivity and PPV of the abPG-SGA decreased to 73% and 41%, respectively, with a AUC of 0.841.

**Conclusions:**

Malnutrition is prevalent in patients with IBD and using malnutrition risk screening tools such as the abPG-SGA may be useful to identify patients who would benefit from further assessment.

## Introduction

Under and over nutrition are frequently observed complications in patients with inflammatory bowel disease (IBD) and are predictors of poor clinical outcomes in IBD including increased rates of infection, longer hospital stays, prolonged recovery time after surgery, and higher healthcare costs.^1,2^ The ESPEN IBD nutrition guideline recommends routine malnutrition screening for patients with IBD using a validated screening tool^3^; however, guidance around such tools was not provided. Despite the recommendation for malnutrition screening, this is performed infrequently in clinical practice.^4,5^ There are several possible explanations for this including lack of IBD validated malnutrition screening tools applicable to both over- and underweight, and limited resources for implementing malnutrition screening in busy clinical settings.^4,6^

In general populations, the subjective global assessment (SGA) is a validated nutrition assessment tool, and was predictive of major outcomes such as hospital length of stay, mortality, and postoperative outcomes.^7^ The European Society of Clinical Nutrition and Metabolism (ESPEN)^8^ has proposed the SGA as a tool to diagnose malnutrition. Two studies have evaluated the association between SGA and clinical outcomes in IBD, with discordant results.^9,10^ One study associated SGA status with length of hospital stay,^9^ and the other study did not find correlations between SGA and hospitalizations, disease complications, or need for surgery.^10^ Nutrition assessment (including SGA assessment) is detailed and resource intensive, and typically conducted by a registered dietitian (RD) to confirm nutrition diagnosis and management. As RD access is a finite resource, it is not possible for all patients with IBD to be referred for nutrition assessment.

Malnutrition risk screening is intended to be a simple and rapid process used to identify patients who may benefit from a referral to a RD for detailed assessment and intervention. Early data demonstrate patients with IBD may be accurately able to self-screen for malnutrition. If true, this could enhance compliance with IBD malnutrition screening recommendations.

Very good levels of agreement between the patient completed Malnutrition Universal Screening Tool (MUST) and the healthcare practitioner completed MUST screen in outpatients with IBD^5,11^ were previously demonstrated, supporting the conclusion that both the patient and healthcare professional completed MUST screens will produce similar results when applied.

The work to date has not however compared the performance characteristics of malnutrition self-screening tools to a health professional completed malnutrition assessment tool such as the SGA.

Accordingly, the aims of this cross-sectional study were to: (1) identify the proportion of patients who meet criteria for malnutrition using SGA criteria^12^ compared to risk of malnutrition using patient reported screening tools. Patient completed screening tools included the abridged patient-generated SGA (abPG-SGA),^13^ Saskatchewan IBD–nutrition risk tool (SaskIBD-NR),^14^ the MUST,^11^ and the Canadian Nutrition Screening Tool (CNST)^15^; (2) to assess the accuracy of patient completed screening tools against SGA criteria; and (3) to compare results between participants with a body mass index (BMI) ≥25 to under 25, between IBD type and in- and outpatients. There is no accepted bedside gold-standard malnutrition assessment tool for patients with IBD. The ESPEN guideline^3^ acknowledged the detrimental outcomes associated with malnutrition, however did not recommend specific malnutrition assessment tools. SGA is a valid tool to diagnose malnutrition in general populations,^8^ and therefore is chosen as the gold-standard malnutrition assessment tool for this study.

## Materials and Methods

### Ethical Considerations

This was a cross-sectional study conducted at 2 hospitals and 2 IBD specialty clinics in Alberta, Canada from May 2017 to March 2018. Approval was obtained from the Health Research Ethics Board at the University of Alberta (Pro00073470) and the University of Calgary (REB17-0890). Written informed consent was obtained from all patients prior to study participation.

### Study Design and Population

All patients were 18 years and older and diagnosed with Crohn’s disease (CD) or ulcerative colitis (UC). Patients were ineligible if they were pregnant or had chronic renal failure on dialysis, chronic pulmonary disease on home oxygen, congestive heart failure with an ejection fraction <40%, were unable to provide informed consent and/or had English language difficulties.

### Data Collection

Participants independently completed the 4 screening tools (ie, abPG-SGA, MUST, CNST, and SaskIBD-NR) and provided demographic data (ie, age, sex, ethnicity, smoking status, and alcohol intake) using an online or paper survey using the REDCAP data capture system. Patients were approached for participation at either an outpatient clinic at the University of Alberta or Calgary or if they were admitted to hospital and agreed to participate by trained research assistants. While a variety of malnutrition screening tools have been reported in the broader literature, the selected tools were of interest due to previous validation in outpatient settings in other disease types. The characteristics and scoring classifications for the 4 malnutrition screening tools are summarized in Table 1. Next, the research assistant completed a height and weight measurement and collected health information (ie, hospitalization history and medication list) from the participant. Body weight was measured to the nearest 0.1 kg. Height was recorded to the nearest 0.1 cm by using a stadiometer.

**Table 1.** Characteristics of nutrition screening tools applied in the study

Screening tool	Parameters					Scoring cutoff
	Weight loss	BMI	Poor appetite/food intake	Food restriction	Others	
abPG-SGA	^a^		^a^		Symptoms affecting food intake, physical activities	Score of 0–5 indicates less risk of malnutrition; score of ≥6 indicates greater risk of malnutrition^15^
MUST	^a^	^a^	^a^		Acutely ill and there has been or is likely to be no nutritional intake for >5 days	Score of 1 indicates medium risk of undernutrition, and score of 2 or more indicates high risk
CNST	^a^		^a^			Two yes answers indicates nutrition risk
SaskIBD-NR	^a^		^a^	^a^		Score of 3–4 indicates medium risk of undernutrition and score of 5 or more indicates high risk

Abbreviations: abPG-SGA, abridged patient-generated SGA; BMI, body mass index; CNST, Canadian Nutrition Screening Tool; MUST, Malnutrition Universal Screening Tool; SaskIBD-NR, Saskatchewan IBD–nutrition risk.

^a^This indicates this characteristic is included in the screening tool.

After this was completed a dietitian, blinded to previously completed data, performed a physical and nutrition assessment used to calculate SGA.

To assess disease activity, the Harvey–Bradshaw Index (HBI)^16^ for CD and the partial-Mayo score^17^ for UC were scored by the treating physician. These scores were then classified into 1 of 4 disease activity categories including remission, mild, moderate, or severe disease.^18^

### Subjective Global Assessment

To diagnose malnutrition using SGA includes an evaluation of the history of recent nutrient intake (ie, adequate, inadequate), weight changes (ie, % of weight loss in past 6 months and past 2 weeks), severity of gastrointestinal symptoms affecting oral intake (ie, eating pain, anorexia, vomiting, nausea, dysphagia, diarrhea, dental problems, feels full quickly, constipation), functional capacity (ie, reduced capacity, fatigue, or loss of function), and a physical examination of body fat, muscle mass, and presence of edema (ie, cachexia, sarcopenia, high metabolic requirement).^12^ Based on this evaluation patients are then classified as well-nourished (A), moderately malnourished (B), or severely malnourished (C). For more information and training on how to perform a SGA please see https://nutritioncareincanada.ca/resources-and-tools/hospital-care-inpac/assessment-sga.

### Sample Size

The primary aim of the current study was to calculate the sensitivity and specificity of the patient-led malnutrition screens compared to SGA for malnutrition. To calculate the sample size an estimated sensitivity of 83% of the screening tools was used as per similar studies^5,11,14^ and a malnourishment prevalence of 30%. With a precision of 0.10, the required sample size was approximately 180 patients using a validated nomogram for estimating the sensitivity and specificity of a medical test.^19^

### Statistical Analysis

All statistical analyses were completed 3 ways: for the sample as a whole and divided by BMI and IBD type. Continuous variable differences between the groups were evaluated with an independent 2-sided *t*-test and categorical differences were evaluated with a Pearson’s chi-square test. Sensitivity, specificity, positive and negative predictive values (PPV and NPV) for each of the patient completed malnutrition screens were calculated and compared to SGA. For these analyses, 2 levels of risk were considered “at risk of malnutrition” or “not at risk of malnutrition” for each screening tool. In addition, a receiver operating characteristic curve (ROC) analysis was used to identify the area under the curve (AUC) and to examine optimal cut-points for each screening tool as a continuous measure compared to SGA. Statistical significance was set at *P* < .05. Statistical analysis was performed using SPSS statistical software (SPSS Inc., Version 26).

## Results

### Prevalence of Malnutrition

Two hundred forty-nine patients were recruited for this study and all patients who were recruited completed the screening tools, height and weight measurements, and dietitian-led SGA assessment. Four patients were excluded from analyses due to missing data or inaccurate diagnosis of IBD. This left a final sample of IBD 149 (61%) CD and 96 (39%) UC patients. Mean age and BMI for all patients with IBD were 42 (SD = 16) years and 26.2 (5.4) kg/m^2^, 51% were male, 16% were current smokers, 82% were outpatients, and 21% had been hospitalized for an IBD-related event in the past 12 months. Patients with a BMI ≥25 kg/m^2^ were significantly older (*P* = .04), more likely to be a smoker, an outpatient, and on biologics (*P* = .01, *P* = .04, and *P* = .003, respectively). Patients with a BMI <25 kg/m^2^ had a higher disease activity (*P* < .05) and less of an appetite (*P* = .03). Fewer patients with UC compared to CD were smokers (*P* = .001), and a greater proportion of UC patients were hospitalized inpatients (*P* = .008), on 5-aminosalicylic acid (*P* < .001) with greater disease activity (*P* = .001). Additional baseline patient demographic and health characteristics are summarized in Table 2. The proportion of patients classified as malnourished using SGA and at risk of malnutrition using patient screening tools are reported in Table 3. Using SGA 23.3% of participants were classified as malnourished while patient completed measures ranged from 26.6% for the MUST to 36.7% for the CNST. For those classified as malnourished using SGA, a significantly higher number had BMIs <25 kg/m^2^ compared to BMIs ≥25 kg/m^2^ (*P* = .005) and had a diagnosis of UC compared to CD (*P* = .007).

**Table 2.** Demographic and clinical characteristics of the study participants

Characteristics	All IBD (*N* = 245)	IBD <25 kg/m^2^ BMI (*n* = 111)	IBD ≥25 kg/m^2^ BMI (*n* = 134)	P*	CD (*n*= 149)	UC (*n* = 96)	P*
Age in years, mean (SD)	41.9 (15.9)	39.7 (16.1)	43.8 (15.5)	**.04**	43.3(15.2)	39.8 (16.7)	.09
Sex, *n* (%)				.23			.29
Male	125 (51.0)	52 (46.8)	73 (54.5)		72 (48.3)	53 (55.2)	
Ethnicity [Caucasian], *n* (%)	193 (87.3)	89 (87.3)	104 (87.4)	.64	119 (91.5)	74 (81.3)	.08
BMI in kg/m^2^, mean (SD)	26.2 (5.4)	21.9 (2.0)	29.9 (4.6)	**<.001**	26.4 (5.1)	26.1 (6.0)	.73
Current smoker, *n* (%)	38 (15.6)	11 (10.0)	27 (20.1)	**.01**	33 (22.1)	5 (5.3)*	**.001**
Current alcohol drinker, *n* (%)	162 (66.4)	77 (70.0)	85 (63.4)	.28	101 (67.8)	61 (63.5)	.56
Outpatients, *n* (%)	201 (82.0)	85 (76.6)	116 (86.6)	**.04**	130 (87.2)	71 (74.0)*	**.008**
Hospitalization history for IBD in past 12 months, *n* (%)	50 (20.7)	27 (25.0)	23 (17.2)	.13	29 (19.7)	21 (22.1)	.66
Treatment of IBD, *n* (%)							
5-ASA	64 (26.1)	28 (25.2)	36 (26.9)	.77	20 (13.4)	44 (45.8)	**<.001**
Immunomodulators	101 (41.2)	41 (36.9)	60 (44.8)	.22	75 (50.3)	26 (27.1)	**<.001**
Biologics	140 (57.1)	52 (46.8)	88 (65.7)	**.003**	97 (65.1)	43 (44.8)	**.002**
Steroids	62 (25.3)	32 (28.8)	30 (22.4)	.25	35 (23.5)	27 (28.1)	.42
Multivitamins	147 (60.0)	65 (58.6)	82 (61.2)	.68	94 (63.1)	53 (55.2)	.22
Disease activity^a^, *n* (%)				**<.05**			**.001**
Remission	135 (55.1)	58 (52.3)	77 (57.5)		90 (60.4)	45 (46.9)*	
Mild	50 (20.4)	19 (17.1)	31 (23.1)		30 (20.1)	20 (20.8)	
Moderate	31 (12.7)	14 (12.6)	17 (12.7)		21 (14.1)	10 (10.4)	
Severe	29 (11.8)	20 (18.0)	9(6.7)		8 (5.4)	21 (21.9)	
Symptoms affecting dietary intake, *n* (%)							
Diarrhea	62 (25.3)	32 (28.8)	30 (22.4)	.25	36 (24.2)	26 (27.1)	.61
No appetite	55 (22.4)	32 (28.8)	23 (17.2)	**.03**	35 (23.5)	20 (20.8)	.63
Pain	57 (23.3)	28 (25.2)	29 (21.6)	.51	36 (24.2)	21 (21.9)	.68
Fatigue	44 (18.0)	21 (18.9)	23 (17.2)	.72	29 (19.5)	15 (15.6)	.45
Nausea	40 (16.3)	19 (17.1)	21 (15.7)	.76	25 (16.8)	15 (15.6)	.81
Early satiety	28 (11.4)	14 (12.6)	14 (10.4)	.6	18 (12.1)	10 (10.4)	.69
Vomiting	21 (8.6)	12 (10.8)	9 (6.7)	.24	13 (8.7)	8 (8.3)	.92

Abbreviations: 5-ASA, 5-aminosalicylates; BMI, body mass index; CD, Crohn’s disease; IBD, inflammatory bowel disease; IQR, interquartile range; UC, ulcerative colitis.

Bold values indicate statistically significant differences between groups.

^a^Disease severity categories using the Harvey Bradshaw Index for CD and the partial-Mayo score for UC.

*Significant differences between subgroups (CD and UC; BMI <25 kg/m^2^ and > 25/kg/m^2^) at *P* < .05 using independent sample *t*-test and chi-squared.

**Table 3.** Malnutrition proportions using registered dietitian-completed SGA and patient completed malnutrition screening tools (*n*, %)

Parameters	All IBD *N* = 245	IBD <25 kg/m^2^ BMI (*n* = 111)	IBD ≥25 kg/m^2^ BMI (*n* = 134)	P*	CD (*n* = 149)	UC (*n* = 96)	P*
Registered dietitian completed							
SGA (B/C)	57 (23.3)	35 (31.5)	22 (16.4)	**.005**	26 (17.4)	31 (32.3)	**.007**
Patient completed							
abPG-SGA	89 (36.3)	50 (45.0)	39 (29.1)	**.01**	50 (33.6)	39 (40.6)	.26
SaskIBD-NR	87 (35.5)	43 (38.7)	44 (32.8)	.34	52 (34.9)	35 (36.5)	.8
MUST	65 (26.6)	48 (43.6)	17 (12.7)	**<.001**	33 (22.3)	32 (33.3)	.06
CNST	90 (36.7)	53 (47.7)	37 (27.6)	**.001**	50 (33.6)	40 (41.7)	.21

Abbreviations: abPG-SGA, abridged patient-generated SGA; BMI, body mass index; CD, Crohn’s disease; CNST, Canadian Nutrition Screening Tool; IBD, inflammatory bowel disease; MUST, Malnutrition Universal Screening Tool; SaskIBD-NR, Saskatchewan IBD–nutrition risk; SGA, subjective global assessment; UC, ulcerative colitis.

Bold values indicate statistically significant differences between groups.

*Significant differences between subgroups (CD and UC; BMI <25 kg/m^2^ and > 25/kg/m^2^) at *P* < .05 using chi-squared.

### Disease Severity, Hospitalization, and Malnourished Status

For all patients with IBD, HBI, and partial-Mayo scores were significantly higher in the “at risk of malnutrition” groups for all patient completed malnutrition screening tools and for SGA (*P* < .05). In addition, hospitalized patients compared to outpatients were much more likely to be classified as malnourished using SGA (57% vs 16%, *P* < .001).

### Accuracy of Screening Tools Compared to SGA Criteria

The accuracy of the screening tools compared to SGA criteria is summarized in Table 4. Overall, the abPG-SGA, had the highest sensitivity at 82.5%, PPV of 52.8%, NPV of 93.6%, and AUC of 0.837 (*P* < .001). The MUST had the highest specificity at 81.8% compared to the abPG-SGA specificity of 77.7%.

**Table 4.** Sensitivity, specificity, PPV, NPV, and area of the ROC curve of malnutrition risk screening tools compared to registered dietitian assessed SGA

	Sensitivity 95% CI	Specificity 95% CI	PPV	NPV	Area under ROC curve
	SGA	SGA	SGA	SGA	SGA
All patients					
abPG-SGA	**82.5** **72.6–92.3**	77.7 71.7–83.6	**52.8** **42.4–63.2**	**93.6** **89.7–97.4**	0.837*
SaskIBD-NR	68.4 56.4–80.1	74.5 68.2–80.7	44.8 34.4–55.2	88.6 83.6–93.6	0.775*
MUST	54.4 41.5–67.3	**81.8** **76.3–87.3**	47.7 35.6–59.8	85.5 80.3–90.6	0.698*
CNST	77.2 66.3–88.1	75.5 69.4–81.7	48.9 38.6–59.2	91.6 87.3–96.0	0.763*
BMI <25 kg/m^2^					
abPG-SGA	**88.6** **78.0–99.1**	75.0 65.3–84.7	**62.0** **48.5–75.5**	**93.4** **87.2–99.7**	0.831*
SaskIBD-NR	71.4 56.5–86.4	**76.3** **66.7–85.9**	58.1 43.4–72.9	85.3 76.9–93.7	0.791*
MUST	71.4 56.5–86.4	69.3 58.9–79.8	52.1 38.0–66.2	83.9 74.7–93.0	0.748*
CNST	85.7 74.1–97.3	69.7 59.4–80.0	56.6 43.3–69.9	91.4 84.2–98.6	0.775*
BMI ≥25 kg/m^2^					
abPG-SGA	**72.7** **54.1–91.3**	79.5 72.0–86.9	**41.0** **25.6–56.5**	**93.7** **88.8–98.6**	0.841*
SaskIBD-NR	63.6 43.6–83.7	73.2 65.0–81.4	31.8 18.1–45.6	91.1 85.2–97.0	0.750*
MUST	27.3 8.7–45.9	**90.2** **84.7–95.7**	35.3 12.6–58.0	86.3 80.1–92.6	0.589
CNST	63.6 43.5–83.7	79.5 72.0–86.9	37.8 22.2–53.5	91.7 86.3–97.2	0.716
Crohn’s disease patients only					
abPG-SGA	**84.6** **70.7–98.5**	77.2 69.8–84.6	44.0 30.2–57.8	**96.0** **92.1–99.8**	0.852*
SaskIBD-NR	69.2 51.5–87.0	72.4 64.5–80.3	34.6 21.7–47.5	91.8 86.3–97.2	0.778*
MUST	57.7 38.7–76.7	**85.2** **78.9–91.5**	**45.5** **28.5–62.4**	90.4 85.1–95.8	0.724*
CNST	**84.6** **70.7–98.5**	77.2 69.8–84.6	44.0 30.2–57.7	**96.0** **92.1–99.8**	0.808*
Ulcerative colitis patients only					
abPG-SGA	**80.6** **66.7–94.6**	**78.5** **68.5–88.5**	**64.1** **49.1–79.2**	**89.5** **81.5–97.4**	0.836*
SaskIBD-NR	67.7 51.3–84.2	**78.5** **68.5–88.5**	60.0 43.8–76.2	83.6 74.3–92.9	0.784*
MUST	51.6 34.0–69.2	75.4 64.9–85.9	50.0 32.7–67.3	76.6 66.2–86.9	0.666
CNST	71.0 55.0–87.0	72.3 61.4–83.2	55.0 39.6–70.4	83.9 74.3–93.6	0.716

Abbreviations: abPG-SGA, abridged patient-generated SGA; BMI, body mass index; CNST, Canadian Nutrition Screening Tool; MUST, Malnutrition Universal Screening Tool; NPV, negative predictive value; PPV, positive predictive value; ROC, receiver operating characteristic; SaskIBD-NR, Saskatchewan IBD–nutrition risk; SGA, subjective global assessment.

Data are presented as percentage. Bolded values highlight the most favorable values in each different patient type.

*Significant at *P* < .001.

### Accuracy of Screening Tools Compared to SGA for BMI and IBD Type

For patients with a BMI <25 kg/m^2^, the sensitivity of the abPG-SGA was highest at 88.6% with a PPV of 62.0%, a NPV of 93.4% with an AUC of 0.831 (*P* < .001). The SaskIBD-NR screening tool had the highest specificity of 76.3% compared to 75.0% in the abPG-SGA.

In patients with a BMI ≥25 kg/m^2^, the sensitivity of the abPG-SGA was highest at 72.7% with a PPV of 41.0%, a NPV of 93.7%, and an AUC of 0.841. The MUST screening tool had the highest specificity at 90.2% in contrast to 79.5% in the abPG-SGA. In patients with a BMI ≥25 kg/m^2^ the ROC analysis identified a cut-point of 3.5, versus the cut-point of 6.0 used in this study, improved the sensitivity of the abPG-SGA to 86.4% (95% CI = 66.7–95.3) with a small decrease in specificity and PPV to 72.3% (63.4–79.8) and 38.0% (25.9–51.8), respectively, while NPV increased to 96.4% (90.0–98.8).

In both IBD subtypes (Table 4), the abPG-SGA had the highest level of sensitivity at 84.6% for CD and 80.6% for UC with PPVs of 44.0% and 65.1%, NPVs of 96.0% and 89.5%, and AUCs of 0.852 and 0.836 (*P* < .001), respectively.

## Discussion

This multicenter study is the first to assess the performance characteristics of various patient-led malnutrition screening tools compared to SGA for malnutrition in IBD. The novelty of the current study is the comparison of multiple patient completed screening tools to identify the tool with the highest sensitivity, specificity, PPV, NPV, and AUC to detect risk of malnutrition in patients with IBD separated by IBD type and BMI categories. Prevalence of malnutrition using SGA criteria was 23.3% overall and varied between patient disease and BMI categories from 16.4% in patients with a BMI ≥25 kg/m^2^ to 32.3% in UC patients; more patients were classified as malnourished who had a BMI <25 kg/m^2^ and who had UC. The higher prevalence of malnutrition observed in UC patients may in part be explained by a higher proportion being hospitalized inpatients with greater disease severity; risk of malnutrition was positively associated with higher disease severity for all the patient completed tools (*P* < .05). In this population, we concluded the patient-led abPG-SGA appeared to be superior to the other tools to identify patients at risk of malnutrition in need of further assessment. The CNST may also be a useful tool in inpatients versus outpatients (Supplementary Table 1) and further studies comparing the sensitivity and specificity of the abPG-SGA to the CNST for inpatient settings are warranted.

Similar to our study, data in oncology patients demonstrates high sensitivity (80%–94%) and good specificity (72%–78%) of the abPG-SGA for detecting malnutrition.^13,20^ The abPG-SGA eliminates the physical examination, disease/condition and metabolic demand assessment components of the traditional SGA and long-form PG-SGA, but retains the medical history component (weight history, food intake, nutrition impact symptoms as well as activities and function). While the performance characteristics of the patient-led abPG-SGA compared to SGA has not been determined previously in patients with IBD, a Chinese study in 78 patients demonstrated that fat free mass indices negatively correlated with long-form PG-SGA scores and disease activity.^21^

For the patient completed screening tools the patient-led abPG-SGA had the highest level of sensitivity to detect risk of malnutrition in patients with BMI <25 kg/m^2^ (88.6%); however sensitivity decreased to 72.7% in those with a BMI ≥25 kg/m^2^. Changing the cut-point of the abPG-SGA to 3.5 from 6 increased sensitivity to 86.4% with only a small decrease in specificity (−7.2%) in the higher BMI group. Using these criteria in those with a BMI ≥25 kg/m^2^, the abPG-SGA only misclassified 3 patients as not being at risk of malnutrition who had a SGA B or C, and identified 31 patients as at risk of malnutrition who were classified as well-nourished, SGA A. Misclassification of risk may either lead to under-recognition of malnutrition risk and failure to refer to a nutritional professional for timely and detailed malnutrition assessment and therapy, or increased unnecessary RD referrals increasing healthcare costs.

Study strengths included the sample size, multicenter recruitment, and use of multiple nutrition screening and assessment methods to identify risk and prevalence of malnutrition. Limitations include that the study population was limited to 2 hospitals and 2 outpatient IBD tertiary Canadian centers, was largely Caucasian, and may not reflect the age, disease type, patient status, or BMI characteristics of other populations. It remains to be seen whether these tools will have similar test characteristics in different populations of patients, including a cohort representing only hospitalized patients, and this requires further evaluation. Furthermore, we have not addressed the Global Leadership Initiative on Malnutrition (GLIM) nutrition assessment criteria in this manuscript. The nutrition community has expressed concerns about a lack of gold-standard nutrition assessment tool that can be applied at the bedside to identify malnutrition in IBD. The GLIM criteria offer a promising solution to this clinical gap. The GLIM criteria include both phenotypic (unintentional weight loss defined as >5% within the past 6 months, or >10% beyond 6 months; low BMI and loss of muscle mass) and etiologic (reduced dietary intake or any reported chronic GI symptoms that impacted food digestion or absorption, or presence of inflammation) criteria for the diagnosis of malnutrition and this combined approach may guide nutrition interventions and expected outcomes. While the GLIM criteria concepts are encouraging as a malnutrition diagnostic tool, as they consider dietary, clinical and muscle measures, these have not been validated in general clinical populations, or in IBD. Moreover, a hierarchy for determining the components to include in the GLIM assessment has not been provided. Further validation of the GLIM is required before it can be considered as the gold standard from which to diagnose malnutrition.

## Conclusion

Given the moderate to high prevalence of malnutrition in patients with IBD, patient self-screening for risk of malnutrition is a promising approach to identify high risk patients who may benefit from malnutrition assessment and intervention by a nutrition care professional. Incorporating patient completed malnutrition screening tools into routine clinical practice may reduce the burden of malnutrition and its consequences, without imposing strain on existing limited resources. The abPG-SGA is a promising malnutrition screening tool in patients with IBD and can be completed by patients in the waiting room or in hospital. Future research directions include the cost–benefit analysis of using malnutrition screening tools to identify malnutrition risk, and ability of these tools to predict IBD-related clinical outcomes.

## Supplementary Material

otab043_supp_Supplementary_Table_S1

## Data Availability

Study data were collected and managed using research electronic data capture tools (REDCAP) hosted at the University of Calgary Clinical Research Unit. REDCAP is a secure web-based application designed to support data capture for research studies, providing: (1) an intuitive interface for validated data entry; (2) audit trails for tracking data manipulation and export procedures; (3) automated export procedures for seamless data downloads to common statistical packages; and, (4) procedures for importing data from external sources.
