# Supplementary material for: Using Patient Completed Screening Tools to Predict Risk of Malnutrition in Patients With Inflammatory Bowel Disease
Source: Crohns Colitis 360. 2021 Jul 7;3(3):otab043. doi: 10.1093/crocol/otab043 (PMC9802362; doi:10.1093/crocol/otab043)
Supplement: otab043_supp_Supplementary_Table_S1 [file otab043_supp_supplementary_table_s1.docx]

**Supplemental Table 1.** Sensitivity, specificity, PPV and NPV of screening tools compared to SGA for both in- and out-patients

|  | **Sensitivity**  **95%CI** | **Specificity**  **95%CI** | **PPV** | **NPV** | **Area under ROC curve** |
| --- | --- | --- | --- | --- | --- |
|  | **SGA** | **SGA** | **SGA** | **SGA** | **SGA** |
| **Outpatients (n=201)** | | | | | |
| abPG-SGA | **71.9**  **53.3-86.3** | 85.2  78.9-90.2 | **47.9**  **37.6-58.4** | **94.1**  **90.2-96.5** | **0.839*** |
| SaskIBD-NR | 62.5  43.7-78.9 | 81.1  74.3-86.7 | 38.5  29.3-48.5 | 92.0  87.9-94.7 | 0.777* |
| MUST | 50.0  31.9-68.1 | **87.6**  **81.6-92.1** | 43.2  31.0-56.4 | 90.2  86.7-92.9 | 0.697* |
| CNST | 65.6  46.8-81.4 | 81.7  75.0-87.2 | 40.4  31.1-50.4 | 92.6  88.6-95.3 | 0.736* |
| **Inpatients (n=44)** | | | | | |
| abPG-SGA | **96.0**  **79.7-99.9** | 10.5  1.3-33.4 | 58.5  54.3-62.7 | **66.7**  **16.4-95.3** | 0.500 |
| SaskIBD-NR | 76.0  54.9-90.6 | 15.8  3.4-39.6 | 54.3  47.0-61.4 | 33.3  12.5-63.6 | 0.477 |
| MUST | 60.0  38.7-78.9 | **27.8**  **9.7-53.5** | 53.6  42.9-63.9 | 33.3  17.1-54.8 | 0.524 |
| CNST | 92.0  74.0-90.0 | 21.5  6.1-45.6 | **60.5**  **54.2-66.5** | **66.7**  **29.0-90.7** | **0.565** |

Data are presented as percentage. Bolded values highlight the most favorable values in each different patient type. *Significant at p<0.001.

**Abbreviations:** *SGA* subjective global assessment; *abPG-SGA* abridged patient generated-SGA; *SaskIBD-NR* Saskatchewan IBD- Nutrition Risk; *MUST* Malnutrition universal screening tool; *CNST* Canadian nutrition screening tool; *PPV* positive predictive value; *NPV* negative predictive value; *ROC* receiver operating characteristic
